# Dr. Robert Liston’s Elements of Surgery

**Published:** 1842-10-01

**Authors:** 


### BIBLIOGRAPHICAL NOTICE.

*Elements of Surgery:* By ROBERT LISTON, Surgeon to the North-London Hospital, &c. &c. *From the Second Edition, with copious Notes and Additions:* By SAMUEL GROSS, M. D., Professor of Surgery in the Louisville Medical Institute, &c. *Illustrated with numerous engravings.* Philadelphia: Ed. Barrington & Geo. D. Haswell, 1842. 8vo. pp. 640.

Accident has prevented our noticing this excellent work before. The "Elements" are too well known, and too justly appreciated to need any praise on our part. With the "Practical Surgery" of the same author, they form the best surgical manual in the language, in which the actual condition of surgical science is very fairly represented. The omissions of the author have been ably supplied by Professor GROSS, and he has added two well written articles on important subjects—Strabismus and Club Foot. We cannot coincide with the expressions used by Dr. Gross towards the "Médecine

Opératoire" of Velpeau. It is indeed a "vast storehouse of research, to which pompous doctors may resort for ancient lore, and prosing teachers for materials for instruction," (*Preface*, p. 6,) but we dissent from the opinion that "beyond this, it is of comparatively little utility." We think that the practical surgeon may consult almost every page with advantage, and we attach great value to manual details from so distinguished an operator and experienced a surgeon. M. C.

